# A Once Malignant Malady: the Story of Schizophrenia and the Path to Prevention

**DOI:** 10.1192/j.eurpsy.2022.39

**Published:** 2022-09-01

**Authors:** J. Lieberman

**Affiliations:** Columbia University, Psychiatry, New York, United States of America

## Abstract

Schizophrenia is synonymous in the public’s mind with madness. To see someone experiencing a florid psychosis, who has lost the ability to distinguish between the real and the imagined, is to know that you are in the presence of insanity. Schizophrenia has existed for centuries. It’s one of the leading causes of disability in the world, with a lifetime prevalence of about 1 percent of the population. That’s 3.3 million people in the US and 78 million worldwide. Schizophrenia afflicts rich and poor, genders, all races and ethnic groups. Schizophrenia has been subject to many misconceptions, from spiritual affliction to social deviance, psychodynamic conflicts to romanticized notions of iconoclastic creativity. Perhaps, the most significant myth about the disease is that there are no effective treatments or cure. But the reality couldn’t be more different: today’s treatments are effective and often lifesaving. Beginning in the mid-twentieth century, scientific progress has enhanced our understanding of schizophrenia as a brain disorder that disrupts thought, perception, and emotion. Findings of abnormalities in brain structure, biochemical analytes and genetic mutations have revealed its causal mechanisms and have guided the search for effective pharmacologic and psychosocial treatments. This presentation traces the evolution of our social and scientific understanding of Schizophrenia. It will elucidate how science and medicine dispelled the superstition and myth surrounding this ancient malady of the mind and forged a path toward its understanding as a brain disorders and offers affected people, not just humane treatment, but the promise of its ultimate prevention.

**Disclosure:**

No significant relationships.

